# CPX-351 (Vyxeos™) treatment in blast-phase myeloproliferative neoplasm (MPN-BP): real-world experience in 12 consecutive cases

**DOI:** 10.1038/s41408-023-00800-2

**Published:** 2023-02-17

**Authors:** Rimal Ilyas, Kristen McCullough, Talha Badar, Mrinal M. Patnaik, Hassan Alkhateeb, Abhishek Mangaonkar, Animesh Pardanani, Ayalew Tefferi, Naseema Gangat

**Affiliations:** 1 Division of Hematology, Mayo Clinic, Rochester, MN USA; 2 Division of Hematology, Mayo Clinic, Jacksonville, FL USA

**Keywords:** Myeloproliferative disease, Myeloproliferative disease

**Dear Editor**,

Blast-phase transformation in myeloproliferative neoplasms (MPN-BP) occurs at rates of 3.9, 2.6, and 9.3%, after median follow-up periods of 8.2, 9.9, and 3.2 years, in polycythemia vera (PV), essential thrombocythemia (ET), and primary myelofibrosis (PMF), respectively [1–3]. A Mayo Clinic and University of Florence, Italy collaborative study of 410 patients with MPN-BP included 248 Mayo Clinic cases in whom treatment details were available [4]; 96% of the patients were dead after a median follow-up of 3.6 months with 1-, 3- and 5-year survival rates of 17, 6, and 4%, respectively; treatment included supportive care (*n* = 121; 49%), chemotherapy (*n* = 103; 42%) with (*n* = 24) or without (*n* = 79) achieving complete remission (CR) or CR with incomplete count recovery (CRi), and allogeneic stem cell transplant (ASCT; *n* = 24;10%); 1- and 3-year survival rates were 66% and 32% for ASCT, 37% and 19% for chemotherapy-treated patients achieving CR/CRi but were not transplanted, and 8% and 1% in the absence of both ASCT and CR/CRi, respectively [4].

Reported chemotherapeutic regimens for MPN-BP included either intensive acute myeloid leukemia (AML)-like induction chemotherapy with 7(cytarabine)+3(daunorubicin/idarubicin), or less intensive treatment with hypomethylating agent (HMA) based combinations with venetoclax (Ven) or ruxolitinib; associated CR/CRi rates were 59% (AML-like induction), 43% (HMA-Ven) and 8% (HMA-ruxolitinib) [4–7]. CPX-351 (Vyxeos™) is a liposomal formulation of daunorubicin and cytarabine and is currently FDA-approved for elderly patients with secondary AML with myelodysplasia related changes and therapy-related AML. In a phase 3 study of older adults with secondary AML, CPX-351 yielded superior remission rates (47.7% *vs* 33.3%) and overall survival (9.56 *vs* 5.95 months) compared to 7 + 3 [8]. The objective of the current study was to obtain preliminary real-world data regarding efficacy and safety of frontline treatment with CPX-351, in patients with MPN-BP.

The current study was conducted under an Institutional Review Board-approved minimum risk protocol that allowed retrospective extraction and analysis of data from MPN-BP patients receiving CPX-351, as first-line therapy, outside the context of a clinical trial at the Mayo Clinic. Study patients were seen between 2018 and 2021 and follow-up was updated in December 2022. Diagnosis of MPN-BP required the presence of ≥20% blasts in either the peripheral blood or bone marrow [9]. All patients received CPX-351, daunorubicin 44 mg/cytarabine 100 mg/m² I/V on days 1, 3, and 5. Bone marrow biopsy was obtained after cycle 1 with response assessed according to the 2017 European Leukemia Net (ELN) criteria [10]. Cytogenetic and molecular studies were performed by conventional karyotype, and next-generation sequencing of a 42-gene panel, respectively. Overall survival was calculated from the time of initiation of CPX-351 to last follow-up or death and evaluated by the Kaplan–Meier method. Analyses were performed using JMP Pro 16.0.0 software package, SAS Institute, Cary, NC.

A total of12 consecutive patients with MPN-BP (median age 63 years, range 46–74; 58% males) received CPX-351 in the upfront setting. Antecedent MPN included PV/post-PV MF (50%), ET/post-ET MF (25%) and PMF (25%). All 12 study patients were *JAK2* mutated; other mutations included *IDH1/2* (*n* = 7), *ASXL1* (*n* = 4), *TP53* (*n* = 3), *TET2* (*n* = 3), *N/KRAS* (*n* = 2) and *SRSF2* (*n* = 1)*, EZH2 (n* = 1*)* and *U2AF1(Q157)* (*n* = 1). Cytogenetics were abnormal in 8 (67%) of patients, with adverse karyotype in 42%. Details on patient characteristics at time of treatment initiation, response rates and subsequent treatments are provided in Table 1.

**Table 1:** Clinical characteristics at time of treatment initiation and outcomes for 12 patients with blast-phase myeloproliferative neoplasm (MPN-BP) treated with CPX-351 in the upfront setting.

Patient No.	Age/sex	MPN type	ELN cytogenetic risk/karyotype	Mutations	Response with duration (months)	Salvage therapy/ response	Consolidation therapy /response	Relapse/ timing/ therapy for relapse	Allogeneic transplant	Toxicity due to CPX-351	Median survival/ dead or alive
#1	61/M	Post PV-MF	Intermediate 47,XY,+8[1]	*JAK2* *IDH2* *BCOR* *BCORL1*	CR 30.2 months		CPX-351 (CR)^a^	No	Yes	Neutropenic Fever	32 months/alive
#2	72/M	Post PV-MF	Intermediate 46,XY[20]	*JAK2* *FLT3-ITD* *IDH2* *NPM1*	CR 8.6 months		HiDAC + midostaurin x2 (CR)^a^	No	Yes	Neutropenic Fever	10 months/alive
#3	76/F	Post ET-MF	Intermediate 46, XX	*JAK2* *ASXL1* *BCOR* *NRAS* *RUNX1* *SRSF2* *PHF5*	CR 13 months		CPX-351	1st relapse Ven/Cyt x2 (CR) 2nd Relapse Ven/Dec x1 (NR)	No	Neutropenic Fever	45 months/dead
#4	47/F	Post ET-MF	Intermediate 46,XX[20]	*JAK2* *IDH2*	PR	Ven/Dec (NR) Ivosidenib (CR)^a^	HiDAC x3 (NR)	No	Yes		24 months/alive
#5	59/M	Post PV-MF	Intermediate 45,XY,der(7;18)(q10;q10)[7]/46,XY[13]	*JAK2* *ASXL1* *CEPBA* *STAG2 TET2*	NR	Ven/Dec x3 (CR)^a^		Yes Post-transplant None	Yes		9 months/dead
#6	65/F	Post ET-MF	Adverse 45,XX,-5,-7,del(12)(p11.2p13),add(15)(q26.1),del(15)(q22q24),+mar[20]	*JAK2* *TP53*	NR	Ven/Dec (CR)		no	No	Neutropenic Fever	3 months/dead
#7	71/M	PMF	Adverse 47–52,XY,+8,+10,+12,+18,+19,+21[cp7]/46,XY[13]	*JAK2* *ASXL1* *EZH2* *KRAS RUNX1 TET2* *IDH1*	NR	Ven/Aza x3 (NR)		n/a	No	Neutropenic Fever	7months/dead
#8	64/M	Post PV-MF	Intermediate 47 XY+8[5]/46 XY[15]	*JAK2* *IDH2*	NR	None		n/a	No	Neutropenic fever	2 months/dead
#9	59/M	Post PV-MF	Adverse 46,XY,add(9)(p13),del(16)(q22)[2]/46,XY,del(11)(q21q23)[1]/4 6,XY[17]	*JAK2* *IDH2* *TP53* *ASXL1*	NR	Enasidenib (NR) Ven/Dec x3 (NR) CLAG-M (NR) Dec (CR)^a^		No	Yes	Nausea/ Diarrhea	25 months/dead
#10	56/F	PMF	Intermediate 46,XX,add(3)(q27)[7]/46,XX[13]	*JAK2*	NR	FLAG-IDA (CR)^a^		Yes Post-transplant Ven/Aza (NR) GO (NR)	Yes		16 months/dead
#11	72/M	Post PV-MF	Adverse 43–45,XY,-4,inv(16)(p13.1q22),-17,+2mar[cp15]/45–46,sl,+ 8[cp2]/45-46,XY,sl,add(5)(q15)[cp3]	*JAK2*	NR	None		n/a	No		5 days/dead
#12	67/F	Post PV-MF	Adverse 44,XX, -7, der (17;18)(q10;p10) [18]/46,XX[2]	*JAK2* *ASXL1* *IDH1* *TET2* *TP53* *U2AF1*	NR	CD123 BiTe Clinical trial (NR)		n/a	No	Neutropenic fever GI toxicity	6 months/dead

Abbreviations: *ELN* European Leukemia Net 2017, *CR* complete remission, *PR* partial remission, *NR* no response, *n. a* not applicable, *PV* Polycythemia Vera, *PMF* Primary Myelofibrosis, *ET* Essential Thrombocythemia, *Ven* Venetoclax, *Dec* Decitabine, *Aza* Azacitidine. *Cyt* Cytarabine, *GO* Gemtuzumab Ozogamicin.

*HiDAC* High-dose cytarabine. *CLAG-M* Cladribine+Cytarabine+GCSF+Mitoxantrone. *FLAG-IDA* Fludarabine+cytarabine+G-CSF+idarubicin, *BiTe* Bispecific T cell engager.

^a^Indicates therapies given prior to AHSCT.

Treatment-emergent toxicity included neutropenic fever in 7 (58%) patients, resulting in death in one patient. Responses following CPX-351 induction therapy included CR in 3 (25%) patients, partial remission (PR) in 1 (8%) patient while none achieved CRi. Patient 1 was a 61-year-old male with antecedent post-PV MF and trisomy 8, *IDH2* and *BCOR* mutated, achieved CR following two cycles of CPX-351 induction therapy. He was bridged to ASCT following one cycle of CPX-351 consolidation and remains relapse-free thirty-two months after leukemic transformation. Patient 2 was a 72-year-old male with antecedent post-PV MF, *FLT3-ITD, NPM1* and *IDH2* mutated with normal karyotype, received one cycle of CPX-351 plus midostaurin and achieved CR. Subsequently, he received two cycles of high dose cytarabine plus midostaurin consolidation followed by ASCT and remained disease-free but succumbed to organizing pneumonia/respiratory failure thirteen months following diagnosis. Patient 3 was a 74-year-old female with antecedent post-ET MF, harboring *ASXL1, NRAS, RUNX1, SRSF2. BCOR, PHF6* mutations, with normal karyotype, achieved CR following induction therapy with CPX-351. Subsequent treatments included CPX-351 consolidation and azacitidine maintenance, following which disease relapsed thirteen months after diagnosis; received Ven plus cytarabine (2 cycles) with achievement of CR for 12 months, followed by second relapse treated with HMA-Ven without response.

Second-line therapy following failure of CPX-351 was pursued in 7 patients and included HMA-Ven (*n* = 4), Ven plus cytarabine (*n* = 1), FLAG-IDA (*n* = 1), and enasidenib (*n* = 1). Three of 5 patients treated with HMA-Ven or cytarabine achieved CR, one of whom was bridged to ASCT, and an additional patient achieved CR and underwent ASCT following salvage therapy with FLAG-IDA. Notably, two patients attained CR with ivosidenib and decitabine plus Ven, respectively, after failure of second line and subsequent therapies, and also proceeded to ASCT (Table 1). At a median follow-up of 11 months (range; 0.2–45 months) from initiation of CPX-351, 10 (83%) patients have died from disease progression (*n* = 7) sepsis (*n* = 2) and respiratory failure (*n* = 1). Median overall survival was 11 months (95% CI, 4.5–35 months) and was longer in six patients that were transplanted (20.5 months *vs* 4.5 months without ASCT, *p* = 0.03).

The current retrospective experience with CPX-351 in patients with MPN-BP was not overtly different than previous observations in similar patients receiving standard 7 + 3 induction or HMA-Ven [4, 5]. The observed CR/CRi rates with 7 + 3 induction and HMA-Ven were 35%/24% and 26%/17%, respectively [4, 5]. Moreover, survival outcomes for patients with AML in CR vs CRi have been previously noted to be similar [11]. A noteworthy observation from the current series is the utility of salvage therapies following failure of CPX-351, which resulted in CR in 5 of 9 (56%) of non-responding patients, of which four patients were bridged to ASCT. We are fully cognizant that such retrospective studies are not conclusive, but it is unlikely that prospective studies will reveal new information that is relevant to survival, which is positively influenced only by ASCT. In other words, the potential value of currently available intensive or less intensive induction chemotherapy for MPN-BP is as a bridge to transplant. However, whether or not bridging is necessary is currently debatable and if so, less intensive therapy (e.g., HMA-Ven) might be more appealing in that regard. The value of AHSCT in securing long-term survival in patients with MPN-BP is further underscored by a recent European Society for Blood and Marrow Transplantation (EBMT), registry-based analysis on 663 transplanted patients with MPN-BP. In the particular study, with median follow-up after ASCT of approximately 5 years, CR, after ASCT was reported in 76% and estimated 3-year survival, was 36% [12]. Importantly, outcome was shown to be superior in the absence of active disease at time of transplant (3-year survival 43% vs 30%) [12]. Nonetheless, the role of pre-transplant bridging including type of chemotherapy in MPN-BP requires further investigation through a prospective controlled study.

## Supplementary information

Checklist
